# The Revolution of Breast Milk: The Multiple Role of Human Milk Banking between Evidence and Experience—A Narrative Review

**DOI:** 10.1155/2021/6682516

**Published:** 2021-02-01

**Authors:** Pasqua Anna Quitadamo, Giuseppina Palumbo, Liliana Cianti, Paola Lurdo, Maria Assunta Gentile, Antonio Villani

**Affiliations:** NICU, HMB “Casa Sollievo della Sofferenza” Foundation, San Giovanni Rotondo, Foggia, Italy

## Abstract

The review recalls the importance of breast milk and deepens the theme of human milk banking, a virtuous reality that is expanding all over the world but is still little known. In the last 15 years, modern biological technologies have crystallized the concept of uniqueness and irreproducibility of human milk, by establishing three new principles: first: human milk: a “life-saving” drug; second: human milk: the best food for preterm infants; and third: human milk: the main component of premature infant care. Our experience teaches us that human milk banking plays many roles that need to be known and shared.

## 1. The Aim of the Topic

This narrative review is an analysis of the literature and experience-driven data on the use of human milk to feed premature infants. This in-depth review is intended to clarify its importance, with the ultimate goal of providing support in our function as clinicians, which is to reduce risks and increase opportunities.

## 2. The Golden Age of Human Milk

It is a scientific priority to deal with breast milk and human milk banking, a virtuous reality which is expanding all over the world but is still little known. Speaking of novelties in terms of breast milk seems an almost paradoxical situation being an entity that has always existed or, as Bruce German (Director of Food and Health, Institute of the University of California) affirms, it is an “ante litteram” super food and we all learn new things every day from milk and that milk as a complete and comprehensive diet is the product of 200 million years of symbiotic coevolution between a mammalian and her infant. The tools of modern science from genomics to molecular anthropology can be leveraged to understand this remarkable process: molecular insights from sugars to oligosaccharides, proteins to encrypted peptides, structures from globules to micelles, intact cells from stem cells to immune cells.

The paradox lies in the fact that, if we analyze the latest scientific findings on the breast milk, the golden age of knowledge concerning the human milk belongs to this day. The first discovery on the stem cells of the breast milk was in 2007 [1]; the proteome of human milk was mapped with 261 proteins never identified before in 2009 [2]; 300 new microRNA molecules were characterized in 2015 [3]. Then, the detection of the milk microbiome, until recently considered sterile, has a specific microbial community that has a crosstalk relationship with the intestinal microbiome which makes it optimal [4–6]. But rather than the feeling of surprise, Bruce German expresses the embarrassment of the entire scientific community with respect to the “*slowness with which the figure of the exceptional nature of mother's milk was acquired*.” More than a novelty, we define it a true revolution of the laws of nature, since in the last 20 years modern technologies in the biological field have crystallized the concept of uniqueness and irreproducibility of human milk by establishing three new principles.

## 3. The New *Principles* of Infant Nutrition

Let us remember that the *principles* are true for all human beings throughout the world; they are immutable, indisputable. Values change over time, but the principles represent what is always true. They are natural laws concerning causes and effects.

The three principles (Table 1) are as follows:

First: human milk: a “life-saving” drug

Second: human milk: the best food for preterm infants

Third: human milk: the main component of premature infant care

These principles [7–14] have totally changed the point of view and the comparison between the types of nutrition has been interrupted so that the advantages of human milk had to be demonstrated with respect to its substitutes, and we rather talk about life and health risks linked to the nonuse of human milk [15–17]. The latter has become a public health issue and not only a lifestyle choice [18].

It is understood that the existence of substitutes for human milk is, however, an important opportunity where conditions for the real nonavailability of breast milk exist.

The definitions have also changed: when we talk about *human milk*, we refer to breast milk and bank milk, which are not in opposition. On the contrary, one promotes the other and must make part of a single integrated system of elements aimed at the common goal of feeding all premature babies in the world with human milk, as a life-saving element [19].

The HMB activities include donor recruitment, collection, transporting, pasteurization, storage, preparation, and administration of milk, but the aims of the milk bank are to promote and support the culture of breastfeeding and milk donations and to provide women's milk to the NICU.

There is strong evidence that breastfeeding and donation complement each other and synergistically contribute to improve child health and survival [20], through the exclusive feeding of all newborns. Milk donation support practices are the most effective method of protecting, promoting, and supporting breastfeeding [21, 22]. The presence of a milk bank in NICU represents a favorable element for breastfeeding and significantly improves both the availability of mother's milk for feeding the premature baby [23–26] and breastfeeding, with higher percentages of nutrition with mother's milk on discharge from NICU [26–29].

This is easily understood if one considers that the presence of a milk bank in NICU results in the activation of standardized methods aimed at increasing the production of breast milk; providing lactation support from educated staff and the positive attitude of an experienced NICU team contribute to successful lactation and breastfeeding even beyond discharge of the infant [30].

This is the experience of many banks like the one in Madrid [28], where there was a higher consumption of own mother's milk during the hospital stay, and the exposure to formula in the first 15 days of life was reduced from 50% to 16.6% and a higher rate of exclusive breastfeeding at hospital discharge (54% vs. 40%). That is also our experience [26] that we shared with a paper where we reported a doubling of the percentages of feeding with mother milk of VLBW and in another very evocative article [27], in its simplicity, of the facilitating role of the milk bank, where significant was the contribution to donation by mothers who gave birth at very low gestational age or at very or extremely low weight.

It is significant that in 5 years of bank activity, 13% of the entire donation came from mothers who gave birth prematurely: about 40% of this percentage refers to women who gave birth at extremely low gestational age < 25 weeks and 35% to those who delivered newborns < 500 grams. The composition data confirm that human milk donated by the mothers of premature infants is particularly valuable, a true biological gem.

But behind the numbers are people with their stories [31], like a mother who gave birth to two ELBW twins at birth weight < 1000 grams. She fed her children exclusively with her own milk for more than 3 years and donated 100 liters of milk.

### 3.1. First Principle: Human Milk—A “Life-Saving” Drug

In recent decades, there has been a significant reduction in infant mortality, which is currently estimated at 4.5 million children in the first year of life with 3 million (that is to say 45%) occurring in the first 28 days [32]. The principal cause of death is no longer infections but prematurity. Many of these deaths are preventable, and of all known approaches, feeding babies exclusively with human milk in the first hours, days, and months of life has the greatest potential impact on child survival and development [33].

There is strong evidence of the protective effect of human milk on the NEC [34–45], which is certainly one of the most important pathologies of VLBW, not because of its incidence but because it is burdened by a high mortality, since half of the preterm with surgical NEC undergoes exitus or can report serious problems of gastrointestinal functionality such as short bowel syndrome.

On the other hand, it has also been demonstrated that the exclusive use of formulas for premature babies is associated with an increased risk of developing NEC compared to breast milk and bank milk [46].

This is also our experience as a milk bank that has completely eliminated the NEC in our NICU [39]. The protective effect of human milk also stands for other complications related to the prematurity [41, 47–49] such as sepsis, ROP, and BPD, but with dose-dependent effects, just like a life-saving drug. Furthermore, the use of human milk is associated with a better neurological [50] outcome thanks to the action of neuroprotection and neuromodulation and the greater sensitivity of a premature brain to the beneficial effects of the HM, in a phase of dendritic and axonal growth and of very rapid synaptogenesis. The best performances, above all cognitive, correspond to anatomical data documented by brain MRI. In the study by Deoni et al. [51], the children fed with BM showed greater white matter development and a positive association between the microstructure of the white matter in many brain regions and the duration of lactation and higher scores of language and visual.

Furthermore, human milk taken in the first month of life has shown clear neurological advantages in anatomical and functional terms.

A direct correlation has been demonstrated between the number of days, in which newborns took their mother's milk (>50% of the ingested milk) in the first month of life, and the volume of gray matter at the end of the 40th week after birth and a better level of neurodevelopment at 7 years of age compared to those who had taken less than 50% of the total breast milk in the first month of life [52].

Moreover, the greater proportion of gray matter in the thalamus and in the basal ganglia of the central structures in cortical connectivity and the efficacy of neural function and the greater volume found in the hippocampus area are well associated with the better performance of memory and learning functions.

In a recent study [53], the authors investigated the influence of breast milk intake during neonatal care in NICU; infants received exclusive breast milk feeds for ≥75% of days of inpatient care, and this was associated with higher connectivity in the fractional anisotropy- (FA-) weighted connectome, compared with the group who had <75% of days receiving exclusive breast milk feeds. In addition, the effect on structural connectivity and tract water diffusion parameters was greater with ≥90% exposure, suggesting a dose effect.

Human milk is considered an optimal “immunonutrition,” and early life is setting the right course for later life.

All these effects recognize in part an indirect genesis, linked to the reduction of the morbidity of prematurity, but in large part, they are directly due to the innumerable nutritional and bioactive factors of the breast milk. This type of analysis is very wide considering the number and specificity of the factors, not all clarified, contained in breast milk. We can report only a few inputs to express the complexity of this system of prevention, nutrition, and coherent development of infants.

More than 1000 types of proteins have been identified, constantly present throughout breastfeeding, in various proportions, each with its own function that can intersect with those of the others in a biological concert, which is little known and plays an important role for the development. For example, nucleotides promote the growth of the immune system and improve the functionality of the gastrointestinal mucosa, favoring the absorption of iron and the colonization of bifidobacteria. Or, LCPUFAs have a concentration that does not change during the 12 months of breastfeeding and their bioavailability in LM is favored by the higher concentration of L-carnitine, a molecule involved in their transport through mitochondrial membranes.

Furthermore, human milk is a privileged site since the nutritional status of the mother relatively influences the composition of the milk, and only the concentration of the vitamins seems to be significantly correlated to the maternal vitamin intake. We also found this aspect in a study [54] where by comparing the clinical-metabolic setting of the donors with the qualitative and quantitative characteristics of the donated milk, this did not change its nutritional features in the presence of clinical-metabolic alterations in the mother. This is an almost “poetic concept,” as if the maternal protective instinct expressed itself also through its milk.

In this regard, we like to mention that a pilot study found that preterm infants exposed to breast milk odour from their own mothers demonstrate a persistent decrease in saliva cortisol levels, which continues after termination of the intervention. This finding may advocate that exposure to own mothers' breast milk odour has a soothing effect on preterm infants. The authors suggest that further randomized controlled studies are needed to evaluate this simple, safe, and inexpensive intervention [55].

Breast milk is a complex mixture of nutrients and biologically active compounds which, together with its microbiota and stem cells, contribute to its many beneficial effects on both mother and child.

The endless list of bioactive factors of breast milk includes *energy factors*: *hormonal factors* that play a role not only in energy balance but also in the metabolic program; *growth factors* that contribute to tissue growth and differentiation; *anti-infective factors* such as a huge amount of secretory IgA and IgM and factors such as lysozyme, lactoperoxidase, lactoferrin, lipoprotein lipase, prebiotics, bifid factor, glycans, oligosaccharides, free fatty acids, and monoglycerides; *other factors*: macrophages, T cells, and lymphocytes and wide variety of cytokines TNF alpha, IL6, IL8, and gamma interferon.

Let us include also stem cells: in one ml of colostrum, there are 5 million stem cells that, marked in mice, have been shown to be absorbed at the gastric level [56]; after, they reach the intestine, work their way through the enterocytes, and enter the circulatory stream to reach all the organs and tissues, where they differentiate into adult cells, including the nervous ones [56], managing to cross the blood-brain barrier as well. Their function is still under investigation. Probably, they represent an internal repair system or perform a function of strengthening and development of organs and tissues, enriching their cellular kit and making them more resistant to diseases of adulthood [57–61]. Therefore, when in literature, we find that human milk can be considered a dynamic biological system specific species; we must believe it. This also explains why all the organizations that deal with international health, WHO, UNICEF, APP, and ESPGHAN, have published the recommendations [10–12] on the feeding of the premature baby. These have been also specified during EXPO 2015 which took place in Milan and which had as its theme the nourishment of the planet (concluding remark: *the first step for feeding the planet is promoting and implementing breastfeeding and human milk donation*).

### 3.2. Second Principle: Human Milk—The Best Food for Preterm Infants

It was from that event that a consensus text was born [62]. It established at the first point that all preterm infants should receive human milk, the second that own mother's milk should be the primary diet, and the third that if MOM is not available or in sufficient quantity, pasteurized donor human milk obtained from a recognized HMB should be used.

Human breast milk was identified as a magic biofluid from ancient times. In Egyptian culture, woman's milk was considered miraculous: a liquid able to heal diseases. Even today, breast milk is undoubtedly the best food for the newborns, since it is able to modulate itself according to their needs from the very early stages of life, in order to ensure their proper development. For this reason, it is also considered the gold standard food for preterm infants, also through appropriate fortification, to be preferred to the formula milk specific for them.

We have recalled that in addition to the classical nutrients (proteins, carbohydrates, lipids, vitamins, and minerals), milk contains several bioactive components, including growth factors, antimicrobial components, and stem cells [56–61], which can integrate in vivo in the tissues (brain, liver, thymus, and kidneys) of the neonate and differentiate in mature cells (for example, in the brain, they transform into neurons, astrocytes, and oligodendrocytes). If we reflect that the premature newborn is growing rapidly, the fastest in the history of the development of a human being, this aspect takes on a particularly relevant meaning.

We can say that fresh breast milk is a life-saving element for premature newborns. The fresh milk of own mother is the most suitable because it is the personalized one for each child and has not undergone any treatment and contains all the biofactors useful for the development and defense of own infant. The protective effect of fresh breast milk on the main complications of prematurity has been amply demonstrated. The evidence on the benefits of donated milk mainly concerns NEC, of which it significantly reduces its incidence, and formula could significantly increase the risks of NEC, while there is currently no clear evidence based on clinical trials on the protective effect against other complications of prematurity [63].

It should be specified that donated milk is not an alternative but rather a bridge to breastfeeding.

It is given to newborns before their mother's milk becomes available, and when the newborn matures the skills for breast attack, it switches to breastfeeding. Feeding with exclusive human milk increases the percentages of breastfeeding on discharge.

Which are the elements that blur the goal of human milk for the premature baby [64]?

- An adequate growth
- The effects of the treatment of donated milk
- Costs

Premature babies that fed on breast milk and even more with bank milk do not grow sufficiently [65, 66]. After all, nature has activated its systems to contain the effects of a devastating occurrence such as premature birth, so much that the milk of mothers of premature babies is different [67], but cannot cope alone with this eventuality.

It is richer in proteins [68] and amino acid composition is closer to the metabolic needs of a developing infant as is preterm and also possesses specific factors such as glucosaminoglycans, substances with antioxidant and anti-inflammatory action that interact with pathogens and compete with them in adhesion to the intestinal wall, behaving like soluble receptors.

Total lipids and caloric content are 20-30% higher in preterm milk, which contains a higher proportion of medium and long chain polyunsaturated fatty acids, LCPUFA omega 3, DHA, and omega 6, important to improve visual and nervous function.

Despite the higher protein content and caloric power of premature breast milk, it is not sufficient to ensure adequate extrauterine growth. On the other hand, adequate growth for the premature infant is essential since protein intake and its effect on anthropometric development and growth speed are closely related to the brain development, and deficiency conditions compromise it [69–71].

This is the rationale of the fortification [72, 73] that is carried out through the supplementation of human milk with polymeric compounds (proteins, lipids, and vitamins) or monomer that provides to increase nutrient intake, in accordance with the guidelines indicated by ESPGHAN.

Another important element regarding the aspect of growth relates to its quality. An increase in lean mass at the end of the correct age was evaluated for late preterm fed with HM [74]. In VLBW, those fed with the formula have an increased fat mass at term [75, 76], while those fed with fortified human milk have a high percentage of lean mass at term [77].

The deposition of lean mass is positively correlated with the neuroevolutionary outcome at 12 months of correct age [78].

As for the treatment of donated milk, obviously this must be pasteurized in order to be safe.

How much impact does the holder method, which is the one commonly used in HMBs around the world, on human milk factors, have? Several works [79–83] have evaluated the effect of pasteurization and many of them are part of a very complete review [83], which in conclusion states that the data indicate that HoP affects several milk components, although it is difficult to quantify the degradation degree.

However, clinical practices demonstrate that many beneficial properties of DHM still persist after HoP and several important factors are not affected by pasteurization [82–89]. The most representative example is that of oligosaccharides. They perform many biological functions (prebiotic effect, modulation of phlogosis, dietary fiber, source of sialic acid and fucose, nutritional effect, and inhibition of bacterial and viral adhesiveness), and their chromatographic pattern is unchanged before and after pasteurization [82, 83].

However, the search for more conservative methods of milk factors is active [90, 91] and the most promising tecnique seems to be rapid high-temperature pasteurization [92–94] (HTST), just 5-15 seconds at 72 degrees, a low-impact and safe pasteurization process.

Another aspect recently introduced in the comparison between the MOM and DHM concerns the intestinal microbiome. In the preterm newborn, the alterations in the early intestinal microbiome predispose to the NEC and to the late onset sepsis [95] (European Perinatal Health Report).

Theodor Escherich, Director of S. Anna Children Hospital to whom you owe the from wet nursing to milk banking in the early 1900s, argued (1884) that the intestinal bacteria of the breastfed neonate were significantly different from those of infants fed in other ways and advocated the life-saving properties of human milk infant nutrition.

The microbial colonization of the neonatal period depends on gestational age, type of delivery [96], and feeding [97].

More *bifidobacteria* and less *Clostridiaceae* have been found in infants fed by MOM compared to DHM, but no significant difference between MOM and DHM with respect to the metabolic and functional profile of the microbiome, which is very different in the formula. The DHM favors a microbiome more similar to the MOM, suggesting a potential effect of DHM to mimic the functionality of the microbiome of those fed with their mother's milk. The practical aspect is that breast milk could potentially be used to customize the donor milk microbiome, and incubating the donated milk with 10% of the mother's milk for 4-8 hours, it would produce a microbial pattern, which is very similar to that of breast milk [98]. This concept is emerging very strongly from metabolomics studies [99].

Different investigations [99–101] evidenced the power of metabolomics as a key technology to improve breast milk's biochemical heterogeneity. The application of metabolomics to HBM is relatively new but particularly interesting because it offers a potent approach to investigate the complex relationships between nutrition and infant's health (nutrimetabolomics). Many results can lead to the comprehensive description of such biofluid and the related effects on breastfed subjects, potentially highlighting the personalized needs of HBM supplementation and/or short- and long-term prevention strategies to optimize offspring health.

### 3.3. Third Principle: Human Milk—The Main Component of Premature Infant Care

In the last decade, the third principle has been fairly understood, so much that the use of DHM has become the standard of care for VLBW when MOM is not available. Today, we know that donor milk from mothers that delivered prematurely has the most adequate composition for preterm infant feeding and that personalized nutrition for premature infants with preterm donor milk is feasible [102]. We know that the fortification of bank milk, especially that of pool of women who have given birth at term, can reduce or cancel the caloric and nutritional gap related to the use of human milk.

DHM banks have been established throughout the world [20]. In a recent systematic review and meta-analysis [103], the census has found 572 milk banks around the world, in 37+ countries, and the authors conclude by stating that the possibility of preserving human milk and promoting donations guarantees an improvement in the health of newborns. In addition, this study shows a clear benefit of breastfeeding or, in its absence, with donated milk and highlights a heterogeneity in the distribution of milk banks between countries and within the same country, particularly in Africa, the Middle East, and Asia, where Muslim populations are dominant.

The countries are very heterogeneous on this focus, both as regards the sociocultural and religious aspects and that of the health system organization and the income, and there are disparities between countries and between north and south of the same country.

Because of the growing interest of the use of DHM in the United States and worldwide, the number of HMBANA (Human Milk Banking Association of North America) milk banks has tripled [20] in the past 10 years (HMBANA, 2017).

The Brazilian model represents the largest network of breast milk donors in the world with 217 milk banks and 126 milk collection points, regulated by public health law that stipulates all the steps required to operate a bank. Moreover, since 1985, Brazil has normalized breastfeeding through its national public awareness campaigns and breast milk donation programs. Since 1985, Brazil's infant mortality rate was by more than two-thirds, 63 to 20 deaths per 1000 births.

Human milk banking has a long history in India. The first human milk bank in Asia was settled in 1989 in Mumbai by Dr. Armida Fernandez, and today, the country has 21 milk banks, mostly in the western region. This number, however, is inadequate to meet the massive need of donors.

In Vietnam, the first donated human milk bank was recently opened in the Hang Hospital for women and children.

In China, the first bank was inaugurated in 2013, and in 3 years, 13 new HMBs were created in the south, east, north, central, and southeast of China and DHM was used not only for preterm infants but also for other sick children [104].

In Africa, there are 70 banks of which 60 are in South Africa and even the human milk banking can be an innovative approach for these developing countries [105].

In Europe, there are 249 active milk banks operating in more than 20 countries, and in Russia, the first human donor milk bank opened in the Scientific Center of Children's Health in Moscow, in 2014. European HMBs are coordinated by the EMBA (European Milk Bank Association) that was launched on 2010.

We agree with the concept that international cooperation and the authorities of the single countries should provide some targeted interventions for the realization of milk banks that, in the last analysis, represent a fortress of health and social justice [104].

In Italy, 38 HMBs are currently operational. They are regulated by law by the Ministry of Health and harmonized by the Italian Association of Milk Banks (AIBLUD).

The first milk bank in Italy was opened at the Meyer in Florence in 1971, and still Tuscany, with 6 centers that network, is the most equipped region.

18 centers have been activated in 20 years, but they cover only 29% of the needs of premature babies [106].

**Figure 1:**
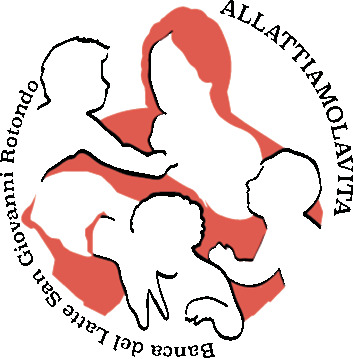
The logo of our HMB (human milk bank).

Our HMB (human milk bank) (Figure 1) is called “Allattiamolavita” which when translated would be “*let us breastfeed the life*,” a title that epitomizes the principles underlying our work which are promotion of breastfeeding, support for life, and donation. Just think of the Hamlet complex [107–110], which, as someone said, makes breast milk a promise of life for cancer patients, as well as being life-saving for premature infants. The logo of our HMB is the stylized version of a famous painting by Guido Reni, currently exhibited in New York museum, where according to the most common interpretation, a sweet damsel through breast milk gives the life to three friends of the artist, who died in a particularly painful moment of his existence.

Our HMB is active since 2010 and has collected 3000 liters of milk, enrolled more than 700 donors, and fed about 600 premature babies [111].

## 4. Economic Aspects of Human Milk Use

We analyzed the economic aspect of human milk's use [104, 112–120]. Professor Meier wants the primacy of this type of studies she has been conducting at the Rush University Medical Center for more than 10 years that, according to her, have not only changed the opinion of the administrators but are widely used in the United States and all around the world, as they first gave monetary value to human milk in NICU [114].

It is important to underline that the administration of donated breast milk, as we have seen, associated with the continuous improvement of neonatal techniques, could significantly reduce the costs of hospitalization and assistance in the short term [104].

In 2016, the York Health Economics Consortium developed the world's first economic model for premature babies and human milk. By feeding an annual population of premature infants with human milk, the UK could reduce healthcare costs by £10.1 million during the first year [115].

The first meta-analysis [116], carried out on more than 100,000 premature babies in the UK and Germany, shows very convincingly that human milk is capable of saving lives and improving both brain development and the newborn's immune system and its use would save society tens and hundreds of millions each year, otherwise needed to cover the health costs and education costs of the children in question.

By administering human milk to premature infants, the UK would increase its GDP by as much as EUR 201 million per year, while Germany would see an increase of EUR 174 million [114–117].

Professor Paula Meier [114], in addition to having shown that the higher the dose of human milk received by the premature or underweight newborn, the better its brain functions in the short and long term, also documented that human milk promotes optimal brain development during adolescence, a period in which the body and brain are subjected to significant stress [118]. This leads to a significant reduction in educational costs that exceed health costs.

The saving is linked to the reduction of expenses for medical care related to the complications of prematurity, but the greatest saving concerns the increase in GDP correlated to the best work and production performances [119].

The additional cost of a donor milk program was small compared with the cost of a NICU hospitalization, in a paper [120] where the authors rated the cost-effectiveness of mother's own milk supplemented with donor milk vs. mother's own milk supplemented with formula for infants of very low birth weight in the neonatal intensive care unit, and that speculate that NICU with greater NEC rates may have greater cost savings.

The economic burden of the human milk banking system is sustainable for states if an operational network is created that includes a reasonable number of HMBs referred to by milk collection centers distributed at all birth points.

If we want to characterize “the investment of a milk bank,” there is an incalculable surplus, which is the human value.

## 5. The Feelings of the Human Milk Donation

We would prefer close by talking about feelings. For this reason, we refer to one of our papers that evaluates the reason of the donation [121]: the altruistic spirit concerned 83% of the answers, in accordance with the literature [122–125]. We asked to describe that experience of the donation, and it was an intense and exciting journey, a precious treasure chest crawling with *spontaneous feelings that bring with them a cultural model to safeguard and spread, made of respect, sharing, and solidarity*.

In conclusion, the many roles of the HMB are as follows (Table 2): carrier of health, feeder facilitator with their own mother's milk, spending review strategy, master of science and research, feeler activator and cultural model to be disseminated. We are convinced that it is only the tip of the iceberg and that many others will be found out.

## Figures and Tables

**Table 1:** 

The new principles of infant nutrition
(i) Human milk: a “life-saving” drug
(ii) Human milk: the best food for preterm infants
(iii) Human milk: the main component of premature infant care

**Table 2:** 

The many roles of human milk banking
Carrier of health
Feeder facilitator with own mother's milk
Breastfeeding promoter
Spending review strategy
Investment with high returns
Master of science and research
Feeler activator
Cultural model to be disseminated

## Abbreviations

HMB: Human milk bank
VLBW: Very low birth weight (<1500 g)
NEC: Necrotizing enterocolitis
NICU: Neonatal intensive care unit
MOM: Mother's own milk
DHM: Donor human milk
HMS: Human milk substitutes
ROP: Retinopathy of prematurity
BPD: Bronchodysplasia
BLUD: Donor human milk bank
AIBLUD: Italian Association of Human Milk Banks
EMBA: European Milk Bank Association
HMBANA: Human Milk Banking Association of North America
GDP: Gross domestic product (economic growth parameter)
FA: Fractional anisotropy
HoP: Holder pasteurization
HTST: Rapid high-temperature pasteurization.
